# Serologic Evidence of Exposure to Highly Pathogenic Avian Influenza H5 Viruses in Migratory Shorebirds, Australia

**DOI:** 10.3201/eid2510.190699

**Published:** 2019-10

**Authors:** Michelle Wille, Simeon Lisovski, Alice Risely, Marta Ferenczi, David Roshier, Frank Y.K. Wong, Andrew C. Breed, Marcel Klaassen, Aeron C. Hurt

**Affiliations:** World Health Organization Collaborating Centre for Reference and Research on Influenza, Melbourne, Victoria, Australia (M. Wille, A.C. Hurt);; Deakin University, Geelong, Victoria, Australia (S. Lisovski, A. Risely, M. Ferenczi, D. Roshier, M. Klaassen);; Commonwealth Scientific and Industrial Research Organisation, Australian Animal Health Laboratory, Geelong (F.Y.K. Wong);; Department of Agriculture and Water Resources, Canberra, Capital Territory, Australia (A.C. Breed);; University of Queensland, St. Lucia, Queensland, Australia (A.C. Breed)

**Keywords:** Australia, avian influenza, clade 2.3.4.4, H5, highly pathogenic, HPAI, influenza A virus, serology, shorebirds, red-necked stint, Pacific black duck

## Abstract

Highly pathogenic avian influenza (HPAI) H5Nx viruses of the goose/Guangdong/96 lineage continue to cause outbreaks in poultry and wild birds globally. Shorebirds, known reservoirs of avian influenza viruses, migrate from Siberia to Australia along the East-Asian-Australasian Flyway. We examined whether migrating shorebirds spending nonbreeding seasons in Australia were exposed to HPAI H5 viruses. We compared those findings with those for a resident duck species. We screened >1,500 blood samples for nucleoprotein antibodies and tested positive samples for specific antibodies against 7 HPAI H5 virus antigens and 2 low pathogenicity avian influenza H5 virus antigens. We demonstrated the presence of hemagglutinin inhibitory antibodies against HPAI H5 virus clade 2.3.4.4 in the red-necked stint (*Calidris ruficolis*). We did not find hemagglutinin inhibitory antibodies in resident Pacific black ducks (*Anas superciliosa*). Our study highlights the potential role of long-distance migratory shorebirds in intercontinental spread of HPAI H5 viruses.

Highly pathogenic avian influenza (HPAI) A(H5N1) viruses of the goose/Guangdong (gs/GD) lineage emerged in domestic birds in China in 1996, causing high morbidity and mortality rates in poultry; subsequent zoonotic spillover in 1997 caused fatal human infections (*1*,*2*). HPAI H5N1 virus reemerged in 2005 and subsequently spread throughout Asia, Europe, and Africa, becoming endemic in parts of Asia and Africa and causing economic losses and human fatalities (*3*,*4*). The role of wild birds in the spread of HPAI H5N1 virus is uncertain, but they probably were not the main culprits in virus spread before 2014 (*3**–**5*). In 2014, and again in 2016, gs/GD lineage HPAI H5Nx virus clade 2.3.4.4 emerged and rapidly spread with wild birds from Asia to Europe, Africa, and North America (*6*–*9*). Unlike other lineages, these 2.3.4.4 clade viruses might cause low morbidity and mortality rates in wild birds, enabling their rapid intercontinental spread through bird migration (*8*,*10*,*11*). Asia, Europe, and Africa continue to report outbreaks of HPAI H5 viruses (*10*). Thus far, Australia, South America, and Antarctica remain free from gs/GD lineage viruses. 

Unlike HPAI viruses, low pathogenicity avian influenza (LPAI) A viruses are part of the natural virodiversity of wild birds. Diverse subtypes and lineages circulate globally, causing no or limited clinical signs of disease (*12*–*14*). Waterfowl (Anseriformes), shorebirds, and gulls (Charadriiformes) are natural reservoirs of LPAI viruses, which have been detected in >100 wild bird species to date.

Natural annual cycles of migratory birds can contribute to the global and rapid spread of gs/GD lineage clade 2.3.4.4 when birds move from northern breeding grounds and spend nonbreeding periods in southern latitudes (*8*). Outbreaks of HPAI H5 virus clade 2.3.4.4 in wild birds and poultry reflect spatial patterns of bird migration, particularly waterfowl migration (*8*,*10*). Australia is part of the East-Asian-Australasian Flyway, and ≈8 million individual birds from 50 shorebird species migrate to the continent each year (*15*–*17*). In addition to Australia, birds in this flyway have stopover sites along the coast of East Asia and breed in Siberia (*17*). 

Shorebirds are involved in the epidemiology of LPAI viruses, particularly in amplifying viruses, as occurred in Delaware Bay, NJ, USA (*18*), but prevalence is generally low and their role in long-distance movement of avian influenza virus (AIV) is unknown (*19*–*22*). One hypothesis is that shorebirds play a limited role in AIV epidemiology and long-distance dispersal, explaining the absence of gs/GD lineage HPAI H5 viruses on the continent of Australia. In contrast to shorebirds, waterfowl in Australia are largely nomadic species that do not migrate outside the Australian-Papuan zone (*23*).

We examined whether long-distance shorebird migrants were exposed to gs/GD lineage viruses. We used the red-necked stint (*Calidris ruficolis*), which uses Australia as a nonbreeding area, as a model migratory species. The red-necked stint has known stopover locations in East and Southeast Asia, where HPAI virus is endemic. We contrasted findings from red-necked stints with those from the resident Pacific black duck (*Anas superciliosa*), a nonmigratory dabbling duck believed to be a natural reservoir for LPAI virus in Australia. 

## Materials and Methods

### Ethics Statement

We received study approval from Deakin University Animal Ethics Committee under permit nos. A113-2010, B37-2013, and B43-2016; and from the Wildlife Ethics Committee of South Australia under permit nos. 2011/1, 2012/35, and 2013/11. The Australian Bird Banding Scheme approved catching and banding procedures under authority nos. 2915, 8000, and 8001. We obtained fauna and research permits from all relevant jurisdictions. The University of Melbourne Biochemistry & Molecular Biology, Dental Science, Medicine, Microbiology & Immunology, and Surgery Animal Ethics Committee approved ferret infections in accordance with the National Health and Medical Research Council code of practice for the care and use of animals for scientific purposes under project license no. 1714183.

### Species and Sample Collection

We targeted mixed flocks of shorebirds for capture with cannon nets as part of a long-term ringing scheme. Since 2011, these birds also have been used for avian influenza surveillance (*24*). Red-necked stints consistently are captured in large numbers during October–March each year, predominantly in the state of Victoria. We also opportunistically collected samples from Western Australia, Northern Territory, and Queensland as part of ringing expeditions. Samples from these locations are not central to the long-term avian influenza surveillance project. Because the red-necked stint is in Australia during October–March, we analyzed and reported data for this species by using the austral summer season. We captured resident Pacific black ducks by using either baited funnel walk-in traps (*25*) or mist nets. We deployed walk-in traps on shorelines and baited them with a seed mix. We set these traps before dawn and operated them during the day; at night, we left traps open so birds could enter and leave freely. To capture waterbirds at night, we erected mist nets on poles above the water surface. We collected most samples from the state of Victoria but also collected samples from South Australia and New South Wales.

After capture, we individually banded all birds with a metal ring with a unique identifier and collected <200 μL of blood from the brachial vein by using the Microvette 200 Z (Sarstedt, https://www.sarstedt.com) capillary blood collection system. We released all birds after banding and collecting blood samples. We stored blood samples at 4°C–8°C until we separated serum by centrifugation 12–24 hours after sampling. We collected 1,531 serum samples from red-necked stints and 394 serum samples from Pacific black ducks for this study.

### General AIV Immunity

We screened serum samples for nucleoprotein (NP) antibodies to ascertain general AIV seroprevalence. We assessed NP antibodies by using a commercially available ELISA, MultiS-Screen Avian Influenza Virus Antibody Test Kit (IDEXX, https://www.idexx.com), following the manufacturer’s recommendations, where a sample/negative (S/N) ratio of <0.5 indicates a positive result. We considered S/N ratios of 0.5–0.6 inconclusive, although this ratio has been demonstrated to correspond to antibody presence in wild birds (*26*,*27*). We calculated seroprevalence and 95% CI by using the bioconf() function of the Hmisc package in R 3.5.1 (https://www.r-project.org).

### Hemagglutinin Inhibition Assay

After NP antibody screening, we assayed positive and inconclusive serum samples for H5 antibodies by using a hemagglutinin inhibition (HI) assay with 1% vol/vol chicken erythrocytes. We selected 7 contemporary HPAI H5 viruses from different gs/GD lineage clades and 2 LPAI H5 viruses endemic to Australia as antigens (Table). We could only test up to 8 antigens per sample because we could collect only a small volume of serum from red-necked stints; for some samples, we could only test against 4 relevant viruses. 

**Table:** Antigens used to assess exposure of red-necked stints and Pacific black ducks to highly pathogenic avian influenza H5 viruses, Australia*

H5 virus clade†	Strain
HPAI	
1.1.1	A/Cambodia/X0810301/2013(H5N1)
2.1.3.2a	A/Indonesia/NIHRD11771/2011(H5N1)
2.3.2.1b	A/barn swallow/Hong Kong/D10-1161/2010(H5N1)
2.3.2.1c	A/duck/Vietnam/NCVD-1584/2012(H5N1)
2.3.4.2	A/Guizhou/1/2013(H5N1)
2.3.4.4	A/gyrfalcon/Washington/41088-6/2014(H5N8)
2.3.4.4	A/Hubei/29578/2016(H5N6)
LPAI H5	A/duck/Victoria/0305-2/2012(H5N3)
	A/wild bird/Queensland/P17-14428-30-01/2017(H5N1)‡

*All HPAI virus strains were 6:2 recombinant viruses on a PR8 backbone with the multi-basic cleavage site removed. All LPAI strains were gamma-irradiated. HPAI, highly pathogenic avian influenza; LPAI, low pathogenicity avian influenza.  †Clade notation as defined by World Health Organization/World Organization for Animal Health/Food and Agriculture Organization H5N1 Evolution Working Group (*28*). ‡Only used for hemagglutinin inhibition assays for serum samples from Pacific black ducks.

We selected representative H5 viral lineages because of their known spatial and temporal distribution and availability of reference viral antigens, such as those selected by the World Health Organization (WHO) as candidate vaccine viruses (CVVs; http://www.who.int/influenza/vaccines/virus/candidates_reagents/a_h5n1/en/) for pandemic preparedness. WHO’s CVVs are 6:2 recombinant viruses on an A/Puerto Rico/8/1934(H1N1)(PR8) backbone with the multibasic cleavage site removed. The 2 LPAI H5 viruses from Australia were gamma-irradiated antigens. We conducted a hemagglutinin assay on selected antigens to determine virus titer, which we then added to HI plates at a dilution of 4 hemagglutinin units. We treated all NP-positive ELISA field samples with a *Vibrio cholerae* receptor-destroying enzyme (RDE II; Denka Seiken Co., https://denka-seiken.com), then inactivated samples with 1.5% sodium citrate.

We raised control antiserum against all virus antigens, except the LPAI viruses A/duck/Victoria/0305-2/2012(H5N3) and A/wild bird/Queensland/P17-14428-30-01/2017(H5N1), in 6–18-month-old ferrets. In brief, we inoculated ferrets intranasally with 1 mL of virus; at 14 days postexposure, we boosted ferrets by intramuscular delivery of a concentrated dose of the same virus into the hind leg; and at 21 days postexposure, we collected a terminal blood sample. We monitored ferrets’ weights, temperatures, and clinical signs throughout. We used antibodies for all 7 H5 viruses in each assay to measure both homologous titers and cross reaction; we also ran antibodies without virus to assess nonspecific agglutination. We serially diluted all serum samples across assay plates, starting with a titer of 1:20, and calculated specificity of antigen-antibody agglutination (Appendix Table 1).

## Results

### Population Immunity to AIVs

**Figure 1:**
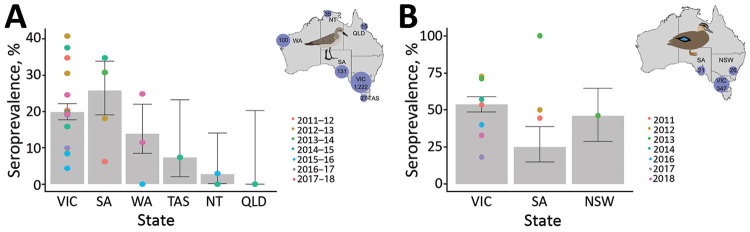
Seroprevalence for nucleoprotein antibodies in red-necked stints and Pacific black ducks, Australia, 2011–2018. A) For red-necked stint, year represents the austral summer period, October–April, when this species has a migratory nonbreeding stopover in Australia. B) For Pacific black duck, year represents calendar year. (No samples were collected in 2015.) Inset maps show the number of samples collected from each species in each state over the course of this study. Error bars represent seroprevalence 95% CIs for each state across all years; color dots represent estimates of seroprevalence at each sampling occasion. NSW, New South Wales; NT, Northern Territory; QLD, Queensland; SA, South Australia; TAS, Tasmania; VIC, Victoria; WA, Western Australia.

During 2011–2018, we collected 1,531 serum samples from red-necked stints, ≈200 samples per year, most from Victoria. Overall, 20% of red-necked stints were seropositive for NP antibodies, with variations among collection years and locations (Figure 1, panel A; Appendix Table 2).

We collected 394 blood samples from Pacific black ducks during 2011–2018. Temporal structure of the data for this species was more variable, with few samples collected during 2015–2017 (Appendix Table 3). We only collected samples from the southeastern states of Australia. Overall, ≈55% of Pacific black ducks sampled were seropositive for NP antibodies. We experienced some variation across sampling events, but average seropositivity was similar across locations (Figure 1, panel B).

### Differences in Exposure to HPAI H5 Virus in Migratory and Resident Birds

**Figure 2:**
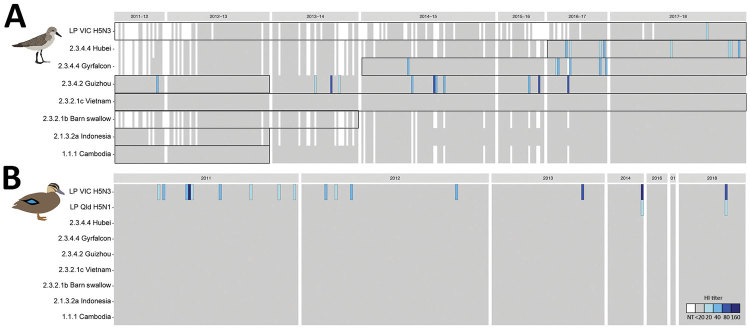
Avian influenza H5 virus hemagglutinin inhibition (HI) antibody patterns, Australia, 2011–2018. A) For red-necked stint, year represents the austral summer period, October–April, when this species has a migratory nonbreeding stopover in Australia. Boxes represent periods of circulation for each strain’s lineage, as determined by genomic sequences (Appendix Table 4). B) For Pacific black duck, year represents calendar year. White indicates untested serum samples; gray indicates a titer <20, the starting titer for this assay; blue indicates hemagglutinin inhibition (HI) antibodies, and shades vary depending on HI titer (20–160). Sample numbers are ordered by collection year and sequentially from left to right in the order in which individual birds were caught. Antigens used in this study are on the y-axis, and abbreviated with relevant clade information; full strain names are available in the Table. NT, no titer. Greater detail on positive samples appears in Appendix Figure 1.

We assayed 307 NP ELISA–positive or –inconclusive serum samples from red-necked stints and 240 from Pacific black ducks for antibodies against H5 viruses by HI assay (Appendix Tables 2, 3). Of HI-positive serum samples, ≈12% were inconclusive by NP ELISA. Because of the small volume of serum collected from red-necked stints, we could assay only 33 serum samples for <4 antigens each (Appendix Table 2). Nonetheless, 23 red-necked stint serum samples contained detectable HI antibodies against >1 of the 7 HPAI H5 virus antigens tested (1.5%, 95% CI 1.0%–2.3%) (Figure 2 panel A). We detected HI antibodies against antigens belonging to clade 2.3.4–derived lineages, specifically 2.3.4.2 A/Guizhou/1/2013(H5N1) (n = 10); 2.3.4.4 A/gyrfalcon/Washington/41088-6/2014(H5N8) (n = 8); and 2.3.4.4 A/Hubei/29578/2016(H5N6) (n = 5). We detected antibodies against A/Guizhou/1/2013(H5N1) during each sampling season, with the exception of birds captured during the 2012–13 austral summer. We detected antibodies against 2.3.4.4 A/gyrfalcon/Washington/41088-6/2014(H5N8) from the 2014–15 austral summer through the 2016–17 austral summer. We also detected antibodies against 2.3.4.4 A/Hubei/29578/2016(H5N6) in samples from the 2016–17 austral summer and the subsequent austral summer. The presence of antibodies against these 2 HPAI virus lineages corresponds with reported circulation of these lineages in Eurasia (Figure 2, panel A). Across all seasons, prevalence of HPAI H5 virus HI antibodies varied from 0.7%–2.1%, with the exception of 2016–17, when 4.5% (95% CI 2.1%–9%) of serum samples contained HI antibodies against HPAI H5Nx virus (Appendix Table 2). 

Overall, HI titers were low; 9/23 serum samples had an HI titer of 20 and 14/23 an HI titer of 40. One serum sample had HI antibodies against the LPAI H5 virus A/duck/Victoria/0305-2/2012(H5N3) (Figure 2, panel A). Overall, no red-necked stint samples were positive for both HPAI and LPAI virus antigens.

Of the 240 Pacific black duck serum samples used for HI assays, none had detectable HI antibodies against any of the HPAI H5 virus antigens (Figure 2, panel B; Appendix Table 3). However, 16 (6%) of the NP–positive serum samples contained HI antibodies that reacted with LPAI H5 virus A/duck/Victoria/0305-2/2012(H5N3), of which 2 samples also had HI antibodies that reacted with LPAI A/wild bird/Queensland/P17-14428-30-01/2017(H5N1) virus (Figure 2, panel B).

## Discussion

Despite intercontinental spread of gs/GD lineage HPAI H5Nx viruses from Asia to Europe, Africa, and North America, we have no evidence that incursions of these viruses have occurred in Australia. A leading hypothesis for the lack of incursion is the absence of Anseriformes birds migrating between Asia and Australia (*23*,*29*). However, millions of shorebirds that are reservoirs for AIV migrate from Siberia to Australia, with stopover sites along the coast of East Asia (*15*–*17*,*29*). We demonstrated that these intercontinental migratory birds have been exposed to gs/GD lineage HPAI H5Nx viruses and have the potential to bring these viruses into Australia. The absence of HI antibodies against gs/GD lineage HPAI H5Nx viruses in a widespread and abundant Anseriformes birds in Australia and the lack of detection during ongoing surveillance activities (*12*) suggest that a virus incursion has not occurred yet. 

Overall, red-necked stints we sampled had low prevalence (≈20%) of NP antibodies, and 1.5% of all serum samples contained HI antibodies against gs/GD lineage HPAI H5Nx virus antigen. We detected the highest seroprevalence of gs/GD lineage HPAI H5Nx virus HI antibodies, 4.5% of all serum samples collected, during the 2016–17 austral summer. A previous study in northwestern Australia during 1992–2009 showed that the red-necked stint and other members of the Scolopacidae family had higher AI virus seroprevalence than other shorebird species tested. Furthermore, H5 HI antibodies were common; 31/260 NP ELISA–positive serum samples had HI titers against HPAI H5N1 virus clade 1 A/chicken/Vietnam/8/2004 (*21*). Similarly, serum samples from ducks during this period also had HI antibodies against this clade but not HPAI H5N1 clade 2 viruses (*21*,*30*). One explanation for the lack of evidence for circulation of HPAI H5N1 virus clade 1 during this time is that exposure to endemic H5 virus strains in Australia produces HI antibodies with broad serologic cross-reactivity (*24*,*31*). 

We found no evidence of cross-reactivity in control antibodies (Appendix Table 1) or cross-reaction in any positive serum samples, including no cross-reactivity between LPAI and HPAI virus antigens. Furthermore, the clades we detected HI antibodies against, 2.3.4.2 and particularly 2.3.4.4, are antigenically distant from previously circulating H5 viruses (*32*), so LPAI virus cross-reactivity is unlikely. Long-distance migratory shorebirds captured in Australia could have been exposed to HPAI H5 virus in the northern hemisphere. Indeed, a red-necked stint tested positive for HPAI H5N6 virus in Hong Kong in 2016 on its southward migration (pers. comm.), strengthening evidence of gs/GD H5Nx virus exposure in this species. 

Studies of ducks in Europe and Mongolia provide further perspective. Gilbert et al. demonstrated the presence of HI antibodies against gs/GD lineage HPAI H5N1 virus in waterfowl in Mongolia. These birds had higher serologic reactivity to HPAI H5 virus than to LPAI H5 virus antigens. That study found limited or no evidence of exposure to HPAI virus antigens in a small representation of waterfowl in Europe (*31*). However, Gilbert et al. conducted the study before the reemergence of gs/GD HPAI virus in Europe. In 2016, Poen et al. demonstrated that 4.2% of birds they surveyed in Europe had HI antibodies against 2.3.4.4 HPAI H5Nx viruses, with much higher prevalence in some species: up to 33% in the mute swan (*Cygnus olor*) and lesser white-fronted goose (*Anser erythropus*) (*33*). Hill et al. reported 80% of mute swans had HI antibodies against HPAI H5N8 virus after several AI outbreaks at a swannery in the United Kingdom (*34*). 

The prevalence of HI antibodies we detected in red-necked stints during the 2016–17 season were comparable to those reported in ducks in Europe, even though red-necked stints have a much lower seroprevalence of AIV in general. Some studies suggest that long-lived avian species, such as swans and seabirds, retain HI antibodies over the course of many years, which could enable expansion of antibody breadth, increasing the number of subtypes against which these birds have antibodies over time (*35*,*36*). An additive effect could explain why mute swans maintained high rates of HPAI H5N8 virus HI antibodies after AIV outbreaks in the United Kingdom (*34*). In contrast, ducks are believed to have relatively poor immune memory and to retain HI antibodies only briefly (*37*,*38*). The expected antibody longevity patterns in shorebirds such as the red-necked stint is unknown, but given the relatively high prevalence of HI antibodies, particularly during 2016–17, we hypothesize that shorebirds retain antibodies longer than ducks. We saw generally low HI titers in serum samples from red-necked stints; 82% of serum samples with detectable HI antibodies had titers <40. Gilbert et al. also reported low titers and hypothesized that tested waterfowl were exposed months or years previously (*31*). Alternatively, the antigens might not have matched the antibodies tested.

Waterfowl species comprise the bulk of avian species sampled in most surveillance schemes for avian influenza and are sampled heavily for H5Nx viruses (*33*,*39*). Shorebirds are central to the ecology of AIV (*13*) but are tested rarely beyond the study from Delaware Bay, NJ, USA, and infection prevalence is much lower (*18*–*22*) than in waterfowl (*13*). However, virology and serology data from Delaware Bay suggest both ruddy turnstones (*Arenaria interpres*) and red knots (*Calidris canutus*) are exposed to a large diversity of hemagglutinin subtypes, and <36% of birds tested had neutralizing antibodies against multiple subtypes, demonstrating host competency (*40*). Furthermore, migratory shorebirds have been implicated in the long-distance movement of LPAI viruses (*41*). Experimental studies have shown limited morbidity and mortality rates associated with infection of some 2.3.4.4 subclades in ducks, demonstrating their ability to act as migratory vectors for these viruses (*11*,*42*–*45*). 

Our understanding of infection and pathogenesis of HPAI H5Nx virus in shorebirds is extremely limited. Experimental exposure of dunlins (*Calidris alpina*) and ruddy turnstones to HPAI H5N1 virus clade 2.2 resulted in contrasting outcomes (*46*,*47*). Immunologically naive dunlins showed clinical signs of infection, and 19/20 birds receiving high or mild doses of the virus died. Birds inoculated with low doses did not get infected (*46*). Ruddy turnstones, in contrast, were not immunologically naive, none died, and birds infected with LPAI and HPAI had similar patterns of viral shedding (*47*). The authors attribute the contrasting results between dunlins and ruddy turnstones to the immunological status of birds, suggesting that cross-immunity played a key role in limiting clinical disease (*47*,*48*). In these experiments, infection rates were the same between birds that were seropositive (subtype unknown) before capture, were first exposed to a LPAI H5 virus strain, or were first exposed to an H7 virus strain, suggesting both homosubtypic and heterosubtypic immunity could play a role in protection (*47*,*48*). However, in ducks, phylogenetic distance between hemagglutinin subtypes plays a role in the degree of the heterosubtypic protective response (*49*), and closely related hemagglutinin subtypes likely drive protection. Red knots were more susceptible to acquiring HPAI H5N1 virus, especially clade 2.2.1, and shed higher viral titers before the onset of clinical disease during the migratory phase because of increased plasma corticosterone (*50*). The authors saw no difference in susceptibility to disease between birds in premigration, fueling, or migratory phases and suggested that, assuming no effect of subclinical exposure on the likelihood of migratory takeoff, red knots could spread HPAI H5 virus through migration (*50*). These studies demonstrate shorebirds could be exposed, survive infection, and potentially disperse HPAI H5 virus over long distances during their migratory phase.

In conclusion, we demonstrated that the long-distance migratory red-necked stint, which spends nonbreeding seasons in Australia, has been exposed to HPAI H5 virus clade 2.3.4.4. We did not detect antibodies against this or other HPAI viruses in our sample of resident Pacific black ducks, suggesting exposure has not occurred in Australia. However, our study highlights the potential for migratory shorebirds to spread HPAI H5 viruses, which should inform future avian influenza surveillance.

Appendix Additional information on highly pathogenic avian influenza H5 viruses in migratory shorebirds in Australia.
