# Supplementary material for: Serologic Evidence of Exposure to Highly Pathogenic Avian Influenza H5 Viruses in Migratory Shorebirds, Australia
Source: Emerg Infect Dis. 2019 Oct;25(10):1903–10. doi: 10.3201/eid2510.190699 (PMC6759277; doi:10.3201/eid2510.190699)
Supplement: Appendix — Additional information on highly pathogenic avian influenza H5 viruses in migratory shorebirds in Australia. [file 19-0699-Techapp-s1.pdf]

Article DOI: https://doi.org/10.3201/eid2509.190699

Serologic Evidence of Exposure to Highly Pathogenic Avian Influenza H5 Viruses in Migratory Shorebirds, Australia

Appendix

Appendix Table 1. Hemagglutinin inhibition assay demonstrating fold differences between homologous and heterologous H5 clade antigens to the corresponding reference H5 clade ferret antibodies used in this study*

	Antibody						
Antigen	*1.1.1 Cambodia*	*2.1.3.2a Indonesia*	*2.3.2.1b Barn Swallow*	*2.3.2.1c Viet Nam*	*2.3.4 Guizhou*	*2.3.4.4 Gyrfalcon*	*2.3.4.4 Hubei*
1.1.1 Cambodia	**1,280**	<20	<20	<20	40	<20	<20
2.1.3.2a Indonesia	<20	**640**	<20	<20	<20	<20	<20
2.3.2.1b BarnSwallow	<20	<20	**1,280**	320	<20	<20	<20
2.3.2.1c VietNam	<20	<20	160	**320**	<20	<20	<20
2.3.4.2 Guizhou	40	<20	<20	<20	**640**	<20	<20
2.3.4.4 Gyrfalcon	<20	<20	<20	<20	<20	**160**	<20
2.3.4.4 Hubei	<20	<20	<20	<20	<20	<20	**80**

*1.1.1 Cambodia, A/Cambodia/X0810301/2013(H5N1); 2.1.3.2a Indonesia, A/Indonesia/NIHRD11771/2011(H5N1); 2.3.2.1b Barn Swallow, A/barn swallow/Hong Kong/D10-1161/2010(H5N1); 2.3.2.1c Viet Nam, A/duck/Vietnam/NCVD-1584/2012(H5N1); 2.3.4 Guizhou, A/Guizhou/1/2013(H5N1); 2.3.4.4 Gyrfalcon, A/gyrfalcon/Washington/41088-6/2014(H5N8); 2.3.4.4 Hubei, A/Hubei/29578/2016(H5N6). Bold indicates homologous antigen-antibody titer.

Appendix Table 2. Summary of red-necked stint serum samples assayed for avian influenza H5 viruses, Australia, 2010–2018*

Year†	No. collected	ELISA–positive‡	ELISA–inconclusive§	Tested by HI assay¶	HPAI H5–positive#	HPAI H5 prevalence, % (95% CI)
2011–12	129	27	8	25	1	0.7 (0.04–4.9)
2012–13	175	55	0	51	0	<0.001
2013–14	274	47	1	43	2	0.7 (0.1–2.9)
2014–15	280	75	0	67	5	1.8 (0.6–4.3)
2015–16	261	15	12	23	2	0.7 (0.1–3.1)
2016–17	177	25	10	30	8	4.5 (2.1–9.0)
2017–18	235	51	16	68	5	2.1 (0.8–5.2)
TOTAL	1,531	295	47	307	23	1.5 (1.0–2.3)

*CI, confidence interval; HI, hemagglutinin inhibition; HPAI, highly pathogenic avian influenza.
†Austral summer, October–April, when the species is in Australia.
‡Sample/negative ratio <0.5, as defined by manufacturer (IDEXX, https://www.idexx.com).
§Sample/negative ratio between 0.5–0.6, demonstrated by Brown et al. (*1*) and Shriner et al. (*2*) to indicate a positive result in wild bird samples.
¶Includes samples for which the ELISA result was positive or inconclusive, and sufficient volume to run at least 3 antigens.
#Positive for $\geq$1 of 7 HPAI H5 antigens used in this study.

Appendix Table 3. Summary of Pacific black duck serum samples assayed for avian influenza H5 viruses, Australia, 2010–2018*

Year	No. Collected	ELISA–positive†	ELISA–inconclusive‡	Tested by HI assay	HPAI H5–positive§	LPAI H5–positive¶
2011	114	60	13	72	0	9
2012	92	65	13	74	0	4
2013	75	48	7	44	0	1
2014	21	12	2	14	0	1
2015	0	0	0	0	0	0
2016	20	8	0	8	0	0
2017	11	2	0	2	0	0
2018	61	20	7	26	0	1
Total	394	215	42	20	0	16

*HI, hemagglutinin inhibition; HPAI, highly pathogenic avian influenza; LPAI, low pathogenic avian influenza.
†Sample/negative ratio <0.5, as defined by manufacturer (IDEXX, https://www.idexx.com).
‡Sample/negative ratio between 0.5–0.6, demonstrated by Brown et al. (*1*) and Shriner et al. (*2*) to indicate a positive result in wild bird samples.
§Positive for any of 7 HPAI H5 antigens used in this study.
¶Positive for either of the 2 LPAI H5 antigens used in this study.

Appendix Table 4. Temporal patterns of circulation of avian influenza H5 virus clades, globally*

	Year									
H5 clade	2010	2011	2012	2013	2014	2015	2016	2017	2018	Total
1	2									2
**1.1.1**	**13**	**1**	**2**							**16**
1.1.2	14	16	29	39						98
1.1-like	1									1
2.1.3.1	3	2						1		6
2.1.3.2	11									11
2.1.3.2a	16	22	4	2	1					45
**2.1.3.2b**	**13**	**10**	**1**				**1**			**25**
2.1.3.2-like	7									7
2.1.3.3	2									2
2.2.1	46	11	1	1	1		1			61
2.2.1.1	10	2				2	2			16
2.2.1.1a	43	6			1		1			51
2.2.1.2	20	99	42	51	63	104	28	1		408
2.2.2	5									5
2.2.2.1	17	15								32
2.3.2.1	1									1
2.3.2.1a	4	50	147	43	32	24	37	61	11	409
**2.3.2.1b**	**7**	**19**	**4**	**4**						**34**
**2.3.2.1c**	**51**	**114**	**51**	**135**	**89**	**241**	**66**	**9**	**16**	**772**
**2.3.4**	**19**	**5**	**3**							**27**
2.3.4.1	6									6
**2.3.4.2**	**15**	**3**								**18**
**2.3.4.4**	**7**	**6**	**7**	**56**	**233**	**403**	**117**	**85**	**6**	**920**
7.2	4	2	7	9	8	2				32
Am-nonGsGD	27	106	42	76	34	25	80	27		417
EA-nonGsGD	12	31	4	4	10	8	4			73
Total	376	520	344	420	472	809	337	184	33	3,495

*Lineage determined by assessments of sequences deposited in GenBank (https://www.ncbi.nlm.nih.gov/genbank). We downloaded metadata for all avian influenza H5 virus hemagglutinin sequences from 2010–2018 (n = 3,495) curated in the Influenza Research Database (https://www.fludb.org). Bold indicates clades included in this study.

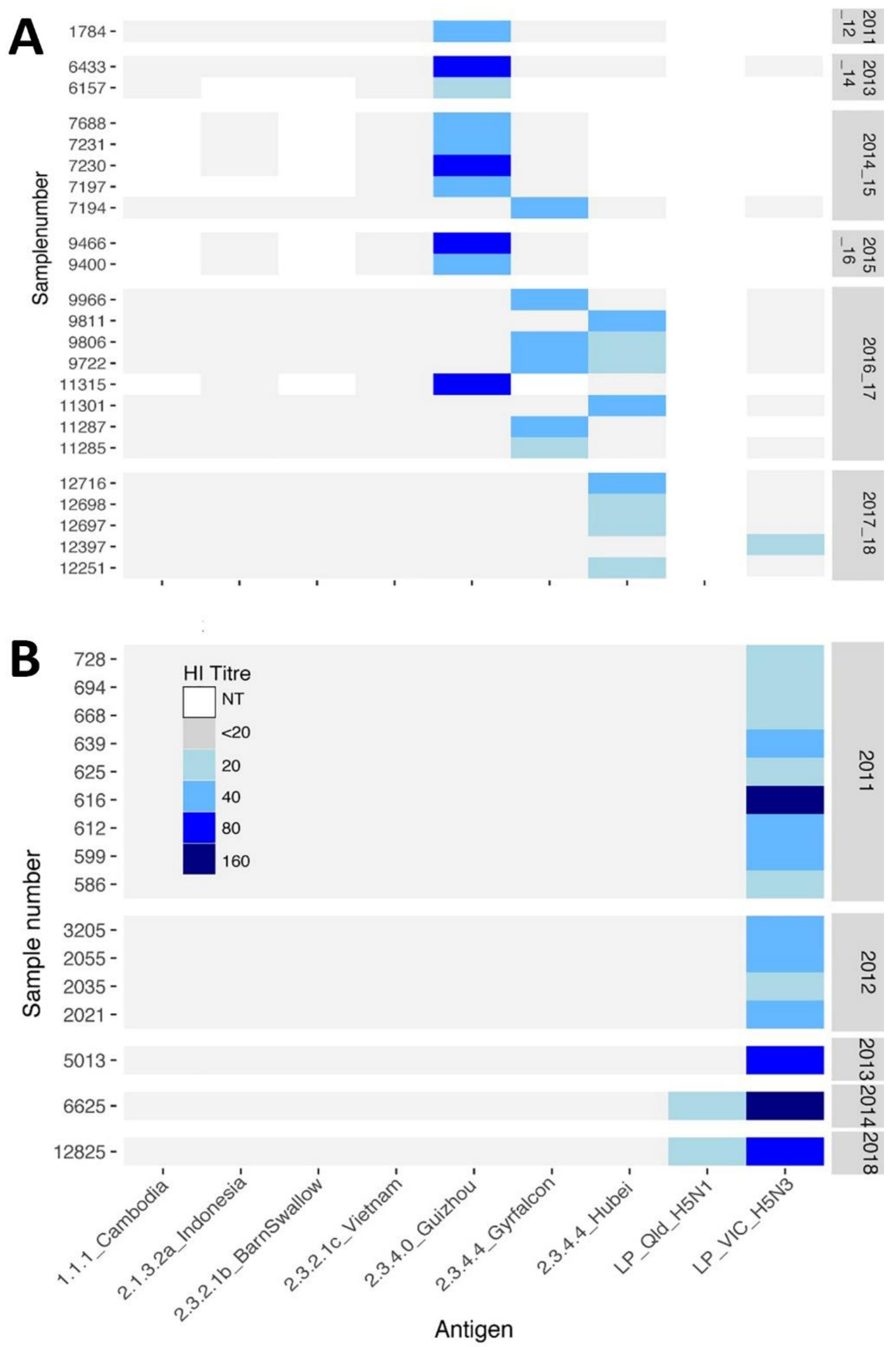

Appendix Figure. Heatmap of serum samples positive for either highly pathogenic or low pathogenic avian influenza H5 virus antigens used in this study. A) Samples from red-necked stints. Year refers to the austral summer (October–April), when this species is in Australia. B) Samples for Pacific black ducks. White indicates serum samples not tested; gray indicates a titer <20, which was the starting titer of this assay; and blue indicates samples containing neutralizing antibodies, shades vary by hemagglutinin inhibition titer (20–160). Sample numbers are ordered by collection year on the y-axis. Antigens used in this study are on the x-axis and abbreviated with relevant clade information.

References

1. Brown JD, Stallknecht DE, Berghaus RD, Luttrell MP, Velek K, Kistler W, et al. Evaluation of a commercial blocking enzyme-linked immunosorbent assay to detect avian influenza virus antibodies in multiple experimentally infected avian species. Clin Vaccine Immunol. 2009;16:824–9. PubMed https://doi.org/10.1128/CVI.00084-09

2. Shriner SA, VanDalen KK, Root JJ, Sullivan HJ. Evaluation and optimization of a commercial blocking ELISA for detecting antibodies to influenza A virus for research and surveillance of mallards. J Virol Methods. 2016;228:130–4. PubMed https://doi.org/10.1016/j.jviromet.2015.11.021
